# Exploring the Neuroprotective Effects of Grape Seed Procyanidins on Amyloid-β-Induced Toxicity in *Caenorhabditis elegans*

**DOI:** 10.3390/foods13233865

**Published:** 2024-11-29

**Authors:** Susana González-Manzano, Begoña Ayuda-Durán, Roberto Martín-Sanz, Lidia Garzón-García, Celestino Santos-Buelga, Ana María González-Paramás

**Affiliations:** 1 Grupo de Investigación en Polifenoles (GIP-USAL), Unidad de Nutrición y Bromatología, Facultad de Farmacia, Universidad de Salamanca, Campus Miguel de Unamuno, 37007 Salamanca, Spain; susanagm@usal.es (S.G.-M.); bego_ayuda@usal.es (B.A.-D.); robertomartinsanzrr@gmail.com (R.M.-S.); lidiagarzon@usal.es (L.G.-G.); paramas@usal.es (A.M.G.-P.); 2 Institute of Biomedical Research of Salamanca (IBSAL), 37007 Salamanca, Spain

**Keywords:** Alzheimer’s disease, *Caenorhabditis elegans*, grape seed polyphenol, amyloid-β, neuroprotection, proteostasis

## Abstract

Alzheimer’s disease (AD), a major neurodegenerative disorder, is characterized by the progressive accumulation of amyloid-β (Aβ) plaques, leading to cognitive decline. Despite the existing treatments, their limited efficacy highlights the urgent need for novel therapeutic strategies. The present study investigates the neuroprotective effects of a grape seed polyphenol extract (GSPE) on transgenic *Caenorhabditis elegans* models specifically expressing human Aβ proteins. The obtained results show that GSPE not only significantly attenuates Aβ-induced paralysis but also extends the lifespan and improves sensory responses in these models, suggesting improved neural function and overall health. Additionally, GSPE treatment reduces proteasomal activity, which could lead to a reduction in the accumulation of misfolded proteins. It also modulates the expression of key genes involved in autophagy and proteostasis, thereby enhancing cellular mechanisms to manage protein aggregation and combat oxidative stress. On the whole, these findings support the potential of grape seed procyanidins (the main components in the extract) to be used as an effective dietary approach to mitigate Alzheimer’s disease pathology through the modulation of critical neuroprotective pathways.

## 1. Introduction

Alzheimer’s disease (AD) is a complex neurodegenerative disorder, which is characterized by the misfolding and aggregation of extracellular amyloid-β (Aβ) plaques and intracellular Tau neurofibrillary tangles (NFTs) in the brain. This is associated with reactive microgliosis, astrogliosis, cerebral amyloid angiopathy, and neuronal loss, and results in the progressive deterioration of cognition and memory [1]. As the disease progresses, patients report difficulties in communication, executive function, recognition of direction, learning capabilities, and cognitive thinking [2].

The exact cause of AD is mostly unknown, but it is thought to be due to a combination of genetic and environmental factors. Risk factors for AD include age; lifestyle; family history; certain medical conditions, such as diabetes and heart disease; and comorbidities [3]. The prevalence of AD is increasing in line with rising life expectancy [4]. It has been estimated that on average, 5.05% of the European population over 60 years of age are affected, accounting for 3.31% in men and 7.13% in women [5]. Currently, there are no effective medical treatments to prevent or stop the progression of AD.

In recent years, there has been a growing interest in the role of diet and lifestyle in the prevention and management of AD. Epidemiological studies have shown that diets rich in anti-inflammatory compounds, such as those found in fruits and vegetables, may lower the risk of developing age-related neurodegenerative diseases, such as Parkinson’s disease and AD [6,7]. Polyphenols, a large group of phytochemicals widely distributed in plant foods, have been proposed as promising preventive and therapeutic agents for AD. In particular, grape seed polyphenol extracts (GSPEs) have been reported to prevent Aβ peptide aggregation and disaggregate preformed misfolded Aβ aggregates in vitro [8,9]. In animal models, several studies have found that different GSPEs are able to reduce amyloid neuropathology and mitigate cognitive deterioration [9,10,11,12]. Nevertheless, whether polyphenols from grape seeds and other sources can prevent or slow down the pathogenesis of the disease in humans or reduce brain pathology in AD patients is not clarified yet.

Grape seeds are a rich source of polyphenolic compounds, namely catechins and procyanidins. Bioavailability studies in humans and animal models have shown that the main circulating forms of polyphenols following the oral administration of GSPE are (epi)catechin and its glucuronide and methylated derivatives [13,14]. These metabolites have been detected in the brains of rats and mice following subchronic dosing [15,16]. Furthermore, studies in a blood–brain barrier (BBB) model (hCMEC/D3 cells) revealed that catechins and their methylated metabolites can be transported across this barrier in a time-dependent manner [17], suggesting that they would be able to reach the brain. Procyanidin oligomers are hardly absorbed, so they have not been significantly detected in plasma. Consequently, they transit to the gut, where they are subjected to degradation by the intestinal microbiota, yielding a series of metabolites that might be subsequently absorbed to be mostly found in plasma as conjugated forms [10].

Monomeric epicatechin has been shown to inhibit Amyloid Precursor Protein (APP) processing and reduce Aβ neuropathology in vivo in a mouse model of AD [18]. In animal models, the phase II metabolite 3′-*O*-methyl-epicatechin-5-*O*-β-glucuronide significantly improved the basal synaptic transmission and maintenance of long-term potentiation [19]. This improvement has been linked to the activation of cAMP response element binding protein (CREB) signaling, which is a key factor in synaptic plasticity essential for learning and memory [19]. The oral administration of epicatechin was also found to decrease ROS and lipid peroxidation and improve memory skills in AD-associated oxidative stress induced by the injection of amyloid-β into the CA1 hippocampal region in rats [20]. Overall, these observations suggest that epicatechin and procyanidins may serve as potential therapeutic or preventive agents for AD [21].

*Caenorhabditis elegans* is a simple multicellular organism that offers numerous advantages for studying the effects of phytochemicals, such as small size, transparency, short lifespan, easy culture, and a high degree of homology and conservation with human pathways, which make it a suitable in vivo model for exploring the mechanisms underlying the bioactivity of natural compounds [22].

The present study aims to explore the neuroprotective effects of a procyanidin-rich extract of grape seeds using the mutant strains of *C. elegans*, namely CL4176 and CL2006 strains, which express the human amyloid-β gene in body wall muscle cells [23,24], and CL2355 strain, which expresses amyloid-β in neurons exhibiting defects in chemotactic behavior [25]. It is, thus, expected to obtain further insights into the effects and mechanisms of action of these compounds that, ultimately, may lead to new strategies for the prevention or treatment of Alzheimer’s disease.

## 2. Materials and Methods

### 2.1. Standards and Reagents

The following standards and reagents were used: ampicillin sodium salt, (-)-epicatechin, yeast extract, nystatin, fluorodeoxyuridine, phosphate-buffered saline (PBS), (FUdR), cholesterol, sodium azide, agar, and benzaldehyde, all sourced from Sigma-Aldrich (Madrid, Spain). Calcium chloride, dimethyl sulfoxide (DMSO), sodium chloride, and a 10% *w*/*v* sodium hypochlorite solution were acquired from Panreac (Barcelona, Spain). Sodium monohydrogen phosphate, potassium dihydrogen phosphate, magnesium sulfate tryptone medium, and potassium monohydrogen phosphate were from Merck (Darmstadt, Germany). Petri dishes with diameters of 35 mm and 60 mm were provided by Brand GMBH (Wertheim, Germany). Peptone and tryptone glucose yeast extract agar were obtained from Fluka Analytical (Madrid, Spain).

### 2.2. Caenorhabditis elegans Strains

The mutant *C. elegans* strains used in this study included CL2355 (dvIs50 [pCL45(snb-1::Abeta 1-42::3’ UTR(long) + mtl-2::GFP] I), CL2122 (dvIs15 [(pPD30.38) unc-54(vector) + (pCL26) mtl-2::GFP]), CL2006 (dvIs2 [pCL12(unc-54/human Aβ peptide 1-42 minigene) + rol-6(su1006)]), and CL4176 (dvIs27[pAF29(myo-3/A-Beta 1-42/let UTR) + pRF4(rol-6(su1006))]). Additionally, the *E. coli OP50* bacterial strain was sourced from the Caenorhabditis Genetics Center at the University of Minnesota (Minneapolis, MN, USA).

Studies in Caenorhabditis elegans do not require approval by the Ethics Committee, as they are free of ethical concerns. European or American regulations on animal welfare only refer to vertebrates, while invertebrates, such as C. elegans or Drosophila melanogaster would be excluded from any prohibition.

### 2.3. Grape Seed Polyphenol Extract (GSPE)

#### 2.3.1. GSPE Preparation

Grape seeds were obtained from drained grapes of cv. Tinta de Toro (2022 harvest) supplied by Rejadorada LLC (San Román de Hornija, Valladolid, Spain), a commercial winery within the PDO Toro (Zamora, Spain). The seeds were manually separated, freeze-dried, and then crushed to obtain a powder.

A 1.5 g of seed powder was added to a 50 mL Falcon^TM^ tube along with 45 mL of methanol. The samples were placed in a shaker for 3 h at 35 rpm and then centrifuged (4000 rpm, 10 min); the supernatant was collected and 45 mL of methanol was added again. The process was repeated twice for 22 h and 5 h of agitation, respectively. The three supernatants were pooled and the methanol was evaporated under vacuum at 30 °C up to a volume of around 20 mL. Four extracts were prepared following this protocol.

In order to separate polyphenols from other compounds in the seed, the obtained extracts were fractionated by molecular exclusion and partition chromatography using Sephadex LH-20. The resin (60 g), reconstituted according to the manufacturer, was suspended in ethanol and poured into a glass column (4 × 53 cm) filled up to a volume of 150 mL. Afterward, 20 mL of the methanolic extract was added to the column and washed with water (450 mL) followed by elution with ethanol (750 mL) at a flow rate of 2 mL/min. The ethanol eluate, containing flavan-3-ols, was collected; ethanol was removed in a rotary evaporator; and the residue was dissolved in water before freeze-drying. After the lyophilization of the four prepared extracts, a total amount of 135 mg of GSPE was obtained and characterized by HPLC-DAD-ESI/MS.

#### 2.3.2. GSPE Characterization

Analyses were performed in a Hewlett-Packard 1200 series liquid chromatograph (Agilent Technologies, Waldbronn, Germany) equipped with a binary pump and a diode array detector (DAD). The stationary phase was a Poroshell 120 EC-C18 column (150 × 4.6 mm i.d., 2.7 μm, Agilent Technologies) thermostatted at 35 °C. The mobile phase consisted of 0.1% (*v*/*v*) aqueous formic acid solution (solvent A) and HPLC-grade acetonitrile (solvent B). The following gradient was established at a flow rate of 0.5 mL/min: 0% B for 15 min, from 0 to 15% B for 10 min, from 15 to 20% B for 10 min, from 20 to 35% B for 10 min, from 35 to 60% B for 10 min, and a final isocratic gradient of 60% B for 5 min. Detection was carried out at 280 nm as the preferred wavelength, and the UV-vis spectra of the peaks were recorded from 220 to 400 nm. MS detection was performed in a 3200 Qtrap mass spectrometer (Applied Biosystems, Darmstadt, Germany) consisting of an ESI source and a triple quadrupole-ion trap mass analyzer. For data acquisition, Enhanced MS (EMS) was used followed by Enhanced Product Ion (EPI) analysis for the fragmentation of the majority ion. Detection was carried out in negative-ion mode (ESI-), and spectra were recorded between *m*/*z* 100 and *m*/*z* 1500. Zero-grade air was used as the nebulizer gas (50 psi) and turbo gas for solvent drying (500 °C, 40 psi), whereas nitrogen served as the curtain (25 psi) and collision gas (medium). Both quadrupoles were set up as unit resolution. The EMS parameters used were as follows: ion spray voltage −4500 V in the negative-ion mode, DP −65 V, EP −10 V, and CE −20 V, while the EPI settings were DP −40 V, EP −8 V, CE −50 V, and CES 0 V.

Compounds were identified from their chromatographic and spectral characteristics (elution order, UV spectra, *m*/*z* ratio, and fragmentation patterns) and comparison with the polyphenol database library made up in our laboratory. Flavan-3-ol quantification was performed from the peak areas recorded at 280 nm by comparison with a calibration curve prepared with epicatechin.

### 2.4. C. elegans Maintenance

Synchronized worm cultures were obtained by allowing gravid hermaphrodites to lay eggs for 2–3 h on fresh plates. Alternatively, synchronization was achieved by subjecting gravid hermaphrodites to a solution of bleach:5 N NaOH (2:1). While eggs are resistant to this treatment, the worms are dissolved in this solution. The resulting suspension was vigorously mixed with a vortex mixer for 1 min and then allowed to rest for an additional minute. This entire process was repeated five times. Subsequently, the suspension was subjected to centrifugation (9500× *g*, 2 min). The resulting pellet, containing the eggs, was washed six times with an equivalent volume of buffer M9 (composed of 3 g KH_2_PO_4_, 6 g Na_2_HPO_4_, 5 g NaCl, 1 mL of 1 M MgSO_4_, and H_2_O to 1 L). In both synchronization methods, once the process was completed, the eggs were transferred onto nematode growth medium (NGM) agar plates, either supplemented with GSPE (100 µg/mL dissolved in DMSO on the plates) or without it. Control plates contained an equivalent volume of DMSO (0.1% *v*/*v*) but no GSPE. The CL2122, CL2355, and CL4176 strains were routinely maintained at 16 °C, whereas the CL2006 strain was propagated at 20 °C on NGM plates seeded with *E. coli* OP50 as a food source.

### 2.5. Paralysis Assay

This assay was conducted as described by Chen et al. [26]. Synchronized CL4176 worms, maintained at 16 °C, were cultured on NGM plates seeded with *E. coli* OP50, either with or without GSPE, at a final concentration of 100 µg/mL. After incubation at 16 °C for 48 h, the temperature was increased to 25 °C to trigger the expression of the Aβ transgene in the muscle cells. The worms were then kept at this temperature for an additional 24 h. Subsequently, the paralyzed worms were scored under a microscope at 24 h and then from 30 to 34 h, with counts performed at 2 h intervals. A worm was considered paralyzed if it exhibited only head bending or showed no movement upon gentle contact. These assays were performed independently three times.

### 2.6. Lifespan Assay

CL2006 synchronized worms on the first day of adulthood were placed on NGM agar plates. These plates were either supplemented with or without GSPE 100 µg/mL. Additionally, the plates contained 150 µM of 5-fluorouracil-2′-deoxyribose (FUdR) to avoid the production of offspring and prevent generational overlap. The worms were cultivated at a temperature of 20 °C, with twenty animals per plate. Each experiment involved a minimum of 100 nematodes. Survivals were transferred to fresh plates every two days; no response of the worms to a gentle touch with a platinum wire was observed, indicating their death. Three independent assays were conducted for all the conditions.

### 2.7. Chemotaxis Assays

Chemotaxis experiments were conducted as described by Dosanjh et al. [27]. The synchronized worms of the transgenic CL2355 strain and its control strain CL2122 were cultured with or without GSPE 100 µg/mL, starting from the egg stage, at 16 °C for 48 h on NGM plates seeded with *E. coli* OP50. Subsequently, the temperature was raised to 25 °C for an additional 36 h to induce Aβ expression in worm neurons. Then, worms were collected and washed twice with M9 buffer, and 50–75 nematodes were carefully placed in the center of a 15 mm diameter NGM plate. In two opposing quadrants of the plate, 1 µL of 1 M sodium azide and 4 µL of 0.5% benzaldehyde in ethanol were applied (“attractant” sites), while in the other two quadrants (“control” locations), 1 μL of sodium azide and 4 µL of 100% ethanol were placed.

The plates were left at 20 °C for 1 h, after which the number of worms in each quadrant was scored. The chemotactic behavior was expressed as the chemotaxis index (CI), defined as [(number of worms in the attractant quadrants − number of worms in the control location)/total number of worms on the plate]. Three independent experiments were conducted, each of them using three replicates.

### 2.8. Proteasomal Activity

Transgenic *C. elegans* CL2006 worms, synchronized and cultured on NGM plates seeded with *E. coli* OP50, were grown at 20 °C with or without GSPE (100 µg/mL) for 120 h. Heat stress was applied at 35 °C for 1 h prior to protein extraction. The worms were then harvested using M9 buffer and centrifuged at 10,000× *g* for 1 min, and the resulting pellet was resuspended in 200 µL of M9. Protein extraction was performed by treating the pellet with 200 µL of NP-40 lysis buffer, adding 10 stainless steel beads (2 mm) to enhance cell disruption. The samples were vortexed vigorously and homogenized seven times using a FastPrep-24 5G system (MP Biomedicals, Irvine, CA, USA) at a speed of 5.5 m/s for 10 s per cycle. Lysates were centrifuged at 13,000× *g* for 15 min at 4 °C, and the protein concentration in the supernatant was quantified using a Nanodrop 2000 spectrophotometer (ThermoFisher Scientific, Waltham, MA, USA). Proteasomal chymotrypsin-like activity was evaluated using the Proteasome 20S Fluorescent Assay Kit (Sigma-Aldrich). The cleavage of the LLVY-R110 fluorogenic substrate by the proteasome produced strong green fluorescence, measured at λex = 485 nm and λem = 520 nm every 10 min for 3 h using a FLUOstar Omega microplate reader (BMG Labtech, Ortenberg, Germany). Specific proteasome activity was determined by subtracting fluorescence signals recorded in the presence of the proteasome inhibitor MG-132 (Z-Leu-Leu-Leu-al; 10 µM final concentration, Sigma-Aldrich) from the overall fluorescence values. Each fluorescence reading was normalized to the total protein content of the respective sample.

### 2.9. Gene Expression Assay

Nematodes synchronized from the egg stage were treated with or without GSPE for 7 days. After treatment, the worms were collected, washed with M9 buffer, and subjected to total RNA extraction using the RNAspin Mini Kit (product number 25050071, Cytiva, MA, USA). Nematode homogenization was performed in a FastPrep-24 5G system at a speed of 5.5 m/s for 10 s, and repeated seven times, with the inclusion of 2 mm stainless steel beads.

For the synthesis of complementary DNA, SuperScript IV VILO Master Mix (Invitrogen) was used at 25 °C for 10 min, and 50 °C and 85 °C for 5 min. The synthesized cDNA was then used to measure the mRNA levels of genes, including *vha-5*, *cpr-5*, *epg-8*, *imp-2*, and *rab-7*, by RT-qPCR using the primers listed in Table 1. The specific target amplification (STA reaction) of cDNA was performed using the QIAGEN Multiplex PCR Kit (Hilden, Germany), with initial heating to 95 °C for 15 min, followed by 20 cycles of 95 °C for 15 s and 60 °C for 4 min. A 1.25 μL aliquot of each cDNA sample was combined with 0.5 μL pooled DeltaGene primers (500 nM), 0.75 μL nuclease-free water, and 2.5 μL multiplex mix. Exonuclease I was then added to remove unincorporated primers and the mixture was incubated at 37 °C for 30 min and 85 °C for 15 min. The pre-amplified cDNA samples were then analyzed by RT-qPCR on a BioMark HD system (Fluidigm, South San Francisco, CA, USA). For these assays, 2.25 μL of each amplified cDNA sample was combined with 2.5 μL of 2X SsoFast EvaGreen Supermix with Low ROX (Bio-Rad) and 0.25 μL of 20X DNA Binding Dye Sample Loading Reagent (Fluidigm). RT-qPCR primer pairs (100 mM) were diluted 1:10 in Tris-EDTA (giving a total volume of 2.5 μL) and mixed with 2.5 μL Assay Loading Reagent (Fluidigm). Each mixture was individually pipetted into the GE 96.96 Dynamic Array™ Integrated Fluidic Circuit (IFC) (Fluidigm) for analysis. Gene expression was analyzed using the comparative 2^−ΔΔCt^ method with the *act-1* housekeeping gene as an internal control [28,29]. This experimental protocol was repeated in four to six independent experiments.

### 2.10. Statistical Analysis

Data analysis was conducted using the SPSS software (version 23.0; SPSS Inc., Chicago, IL, USA). To compare values across multiple groups, an analysis of variance (ANOVA) was applied to assess significant differences between the treated and control groups. For the paralysis assay, chi-squared tests were used to evaluate associations between categorical variables. Kaplan–Meier survival curves were generated for the lifespan assay, and the log-rank test was employed to determine statistical differences. A *p*-value of less than 0.05 was considered indicative of statistical significance in all the analyses.

## 3. Results

### 3.1. Characterization of the Grape Seed Polyphenol Extract (GSPE)

**Figure 1:**
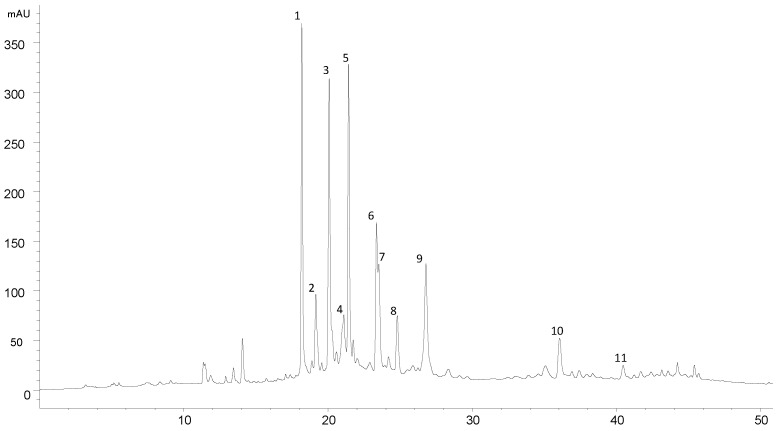
HPLC chromatogram of the prepared GSPE recorded at 280 nm. Peak identities are given in Table 2.

The grape seed extract, prepared as described in Section 2.3.1, consisted of a purified mixture of flavan-3-ol monomers, dimers, and trimers. Figure 1 shows the HPLC profile recorded at 280 nm. Eleven compounds were tentatively identified based on their elution behavior and mass spectra [30] and comparison with our polyphenol database library. In particular, one catechin monomer, five B-type non-galloyled procyanidin dimers, four galloyled procyanidin dimers, and one B-type procyanidin trimer were observed (Table 2).

Flavan-3-ol quantification in the extract was performed by comparison with an epicatechin calibration curve. The results are presented in Table 3. The estimated concentration of total flavan-3-ols in the extract was 162.83 mg epicatechin equivalents/g, with procyanidin dimers representing the predominant form (approximately 68% of the total flavan-3-ols). Galloyled procyanidins accounted for approximately 25% of the total flavan-3-ols.

### 3.2. Paralysis Assays

**Figure 2:**
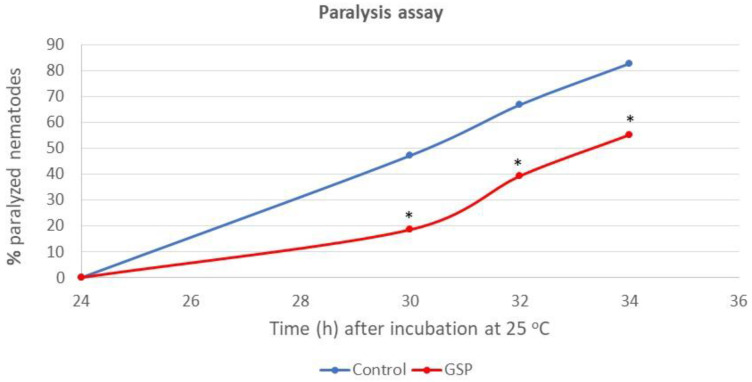
Progression of paralysis rates in the CL4176 strain grown with or without GSPE (100 µg/mL). Data represent the average of three independent experiments (*n* = 100 worms per group). * Statistically significant differences at *p* < 0.01.

These assays were performed in the CL4176 strain, which expresses human Aβ_1–42_ in muscle cells induced by an increase in the temperature from 16 °C to 25 °C. The number of paralyzed worms was scored 24 h after the temperature upshift and further at 30, 32, and 34 h. A significant decrease in nematode paralysis was observed at all the counting times in the treated group (Figure 2).

At the end of the observation time (34 h after thermal shock), the mean percentage of nematodes experiencing total paralysis was 55% in the group treated with GSPE compared to 83% in the not treated worms. These findings suggested that grape polyphenols have the ability to reduce Aβ aggregation and alleviate AD symptoms in CL4176 mutant worms. Comparable effects have been reported for other phenolic compounds and polyphenol-rich extracts. For example, the prenylated flavonols icariin and icariside II have been shown to delay paralysis triggered by Aβ_1–42_ proteotoxicity in the CL4176 strain [31]. Similarly, oleuropein [32], ethyl caffeate [33], and phenolic extracts from *Ginkgo biloba* [34], *Baccharis trimera* [35], Zijuan Pu’er tea [36], and fig peels [37] demonstrated the capacity to mitigate Aβ-induced pathological behaviors in transgenic *C. elegans* models. The anti-aggregation properties of polyphenols against Aβ fibrils and intracellular neurofibrillary tangles are believed to stem from their ability to interfere with the formation of β-sheet structures, a hallmark of Aβ plaques [38,39].

### 3.3. Longevity Assays

Longevity assays were carried out in the CL2006 strain, which, unlike CL4176, expresses, constitutively, Aβ_1–42_ in the cytoplasm of body wall muscle tissues in an age-dependent manner, mimicking the natural progression of AD observed in humans. This results in gradual paralysis, ultimately leading to the nematode’s death [40].

**Figure 3:**
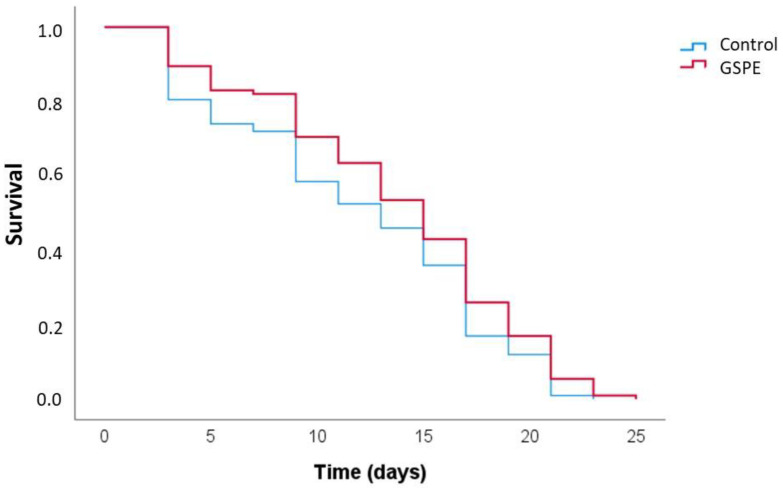
Kaplan–Meier survival curves of the CL2006 worms at 20 °C in the absence (control) and presence of GSPE (100 µg/mL). The plots were obtained from three independent experiments (n = 100 worms/experiment).

The culturing of the CL2006 worms in the presence of GSPE (100 µg/mL) resulted in a higher survival rate, with significant increases in both the mean and maximum lifespan (Figure 3, Table 4). These findings indicated that GSPE has the potential to mitigate the toxic effects of Aβ, delaying the onset of paralysis and ultimately extending the lifespan. Similar results were previously found by our group in studies on CL2006 treated with EC [41], as well as by other authors using different polyphenols or polyphenol-rich extracts [32,42,43], including galloyled flavanols [44].

### 3.4. Chemotaxis Assays

**Figure 4:**
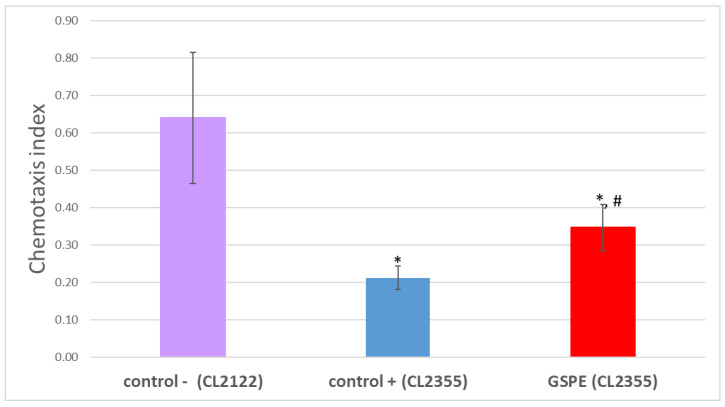
Chemotaxis behavior of the CL2355 strain grown in the absence (positive control) and presence of GSPE (100 µg/mL) after thermal induction (25 °C). The error bars represent the standard deviation. * significant at *p* ≤ 0.05 compared to the negative control; # significant at *p* ≤ 0.05 compared to the positive control.

As shown in Figure 4, the chemotaxis index (CI) was significantly lower in the CL2355 strain (CI = 0.21 ± 0.03; *p* < 0.05), which expresses amyloid-β in neurons following thermal induction, compared to the control strain CL2122 (CI = 0.64 ± 0.17), which is phenotypically similar but that does not express neuronal amyloid-β. The treatment of CL2355 with GSPE (100 µg/mL) significantly improved the worms’ chemotactic response to the attractant benzaldehyde (CI = 0.35 ± 0.06; *p* < 0.05) compared to the untreated CL2355 (positive control). These observations suggest that GSPE protects neurons by decreasing amyloid-β accumulation and subsequently reducing amyloid toxicity.

### 3.5. Proteasomal Activity

**Figure 5:**
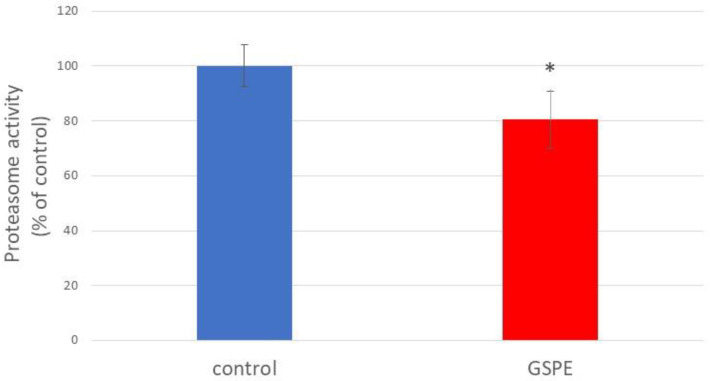
Proteasomal activity in the transgenic CL2006 worms 120 h after treatment with GSPE (100 µg/mL). The results are expressed as a percentage relative to the average value obtained in non-treated worms (control), designated as 100%. Data are indicated as mean ± SD (n = 8). * significant at *p* ≤ 0.05.

Proteasomal activity has been investigated for its role in the observed effects of GSPE. As can be observed in Figure 5, the results showed that the GSPE treatment significantly reduced proteasome activity in the worms. This initially unexpected finding might suggest the existence of lower levels of misfolded and damaged proteins in the treated worms, thus decreasing the need for high proteasomal activity. This effect might result from a direct or indirect effect of GSPE, e.g., the induction of alternative pathways for the elimination of dysfunctional proteins.

### 3.6. Gene Expression Assays

To explore the molecular mechanisms underlying the effects of GSPE, the expression of several genes (i.e., *vha-5*, *cpr-5*, *epg-8*, *imp-2*, and *rab-7*) involved in the autophagy process was analyzed.

**Figure 6:**
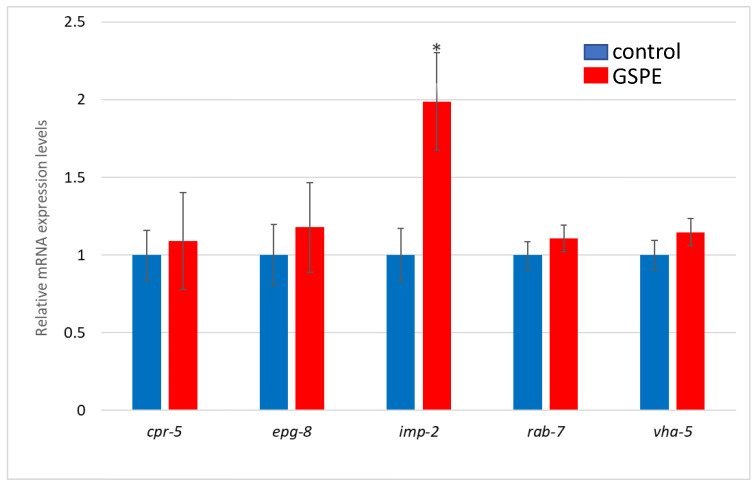
Relative mRNA expression levels of *cpr-5*, *epg-8*, *imp-2*, *rab-7*, and *vha-5* in *C. elegans* CL4176 grown with (treatment: 100 µg/mL GSPE, red bars) or without GSPE (control, blue bars). Measurements were performed by RT-qPCR using *act-1* as a reference. Data represent the mean ± SEM from six independent experiments, with statistical significance set at * *p* < 0.05.

Figure 6 shows the results of the expression analysis of the genes studied in the assays carried out on the *C. elegans* strain CL4176. As it can be seen, only the mRNA levels of the *imp-2* gene are increased in the worms treated with the polyphenolic extract compared to the controls. The significant increase in *imp-2* expression suggests the enhanced efficiency of the autophagosome–lysosome fusion and, consequently, increased autophagy. This may indicate a more effective removal of damaged cellular components in the worms treated with GSPE.

## 4. Discussion

The procyanidin profile in GSPE has been characterized using HPLC-DAD-ESI/MS. A mixture of flavan-3-ol monomers, non-galloyled B-type dimers, galloyled dimers, and procyanidin trimers was identified. Among them, procyanidin dimers accounted for approximately 68% of the total flavan-3-ols content, while galloyled dimers constituted about 25%. The importance of the structure of procyanidins in their efficacy has been highlighted in different studies [13]. Galloyled procyanidins are noted for their enhanced bioavailability and ability to cross the blood–brain barrier (BBB). Once within the brain, these compounds may directly interact with amyloid-β peptides, inhibiting their aggregation or promoting the disaggregation of the existing plaques, which are hallmarks of Alzheimer’s pathology. Serra et al. [13] provided further insights into the bioavailability of procyanidin dimers and trimers, highlighting that despite their size, these molecules can be absorbed in the gastrointestinal tract, albeit less efficiently than monomers. This suggests that even limited systemic availability does not preclude procyanidins from exerting significant biological effects. For example, the metabolic products of procyanidins might also be capable of crossing the BBB and thus contribute to the antioxidant and anti-aggregatory effects observed in GSPE studies. Additionally, the larger procyanidin oligomers, while less bioavailable, may exert local effects in the gut or be metabolized into smaller phenolic acids that are more easily absorbed and could contribute to systemic health benefits, including neuroprotection.

It has long been recognized that the relationship between food and health is intertwined. Several studies have reported that polyphenols, a group of plant secondary metabolites widely distributed in foods, possess neuroprotective properties [45]. This family of compounds shows remarkable multifaceted capabilities, being able to modulate different factors involved in pathological processes, such as reactive oxygen species, metal toxicity, enzyme activity, inflammation markers, apoptosis, signal transduction pathways, ion channeling, or neurotransmitters [46]. Several studies suggested that dietary polyphenols could activate antioxidant pathways, such as Nrf2/HO1, NFκB, MMP, PPAR, HIF-1, and STAT, as well as neurotrophic factors like BDNF, NGF, NT3, and NT4. Polyphenols may also play a role in modulating the immune response by inhibiting pro-inflammatory biomarkers such as CCL17/22, CCR1/2, MIP1 α/1 β, CXCL, IFN-γ, TNF-α, and IL (1β, 6, and 17A) [46,47,48]. The modulation of these pathways would help reduce neurodegeneration. Thus, the direct utilization of polyphenols or their incorporation into functional foods or dietary supplements may have the potential to serve as an approach for the prevention or treatment of neurodegenerative diseases.

Previous studies have demonstrated that various polyphenols confer neuroprotective effects in AD models, particularly in *C. elegans* strains expressing amyloid-β, such as CL2006. For instance, Regitz et al. [42] reported that quercetin ameliorated paralysis in CL2006 by enhancing proteasomal activity and autophagy, thus reducing Aβ aggregation. Similarly, Regitz et al. reported that resveratrol decreased Aβ-induced paralysis through mechanisms that target proteostasis pathways, including the unfolded protein response (UPR) and autophagy [43]. Additionally, Ayuda-Durán et al. [41] found that epicatechin mitigated Aβ toxicity via the modulation of several cellular pathways, including autophagy and proteostasis. Furthermore, Bai et al. concluded that ethyl caffeate attenuated Aβ-induced toxicity through the insulin/insulin-like growth factor-1 signaling pathway in CL4176 [33]. These results suggest that while there are common neuroprotective pathways affected by polyphenols, each specific compound may also activate additional or alternative pathways, contributing uniquely to their neuroprotective profiles.

Improvement in Aβ-induced chemotaxis defects was observed in the CL2355 strain following treatment with polyphenol-rich extracts from *Ginkgo biloba* leaves [34] or flavones isolated from different plant sources [49]. Specifically, a *G. biloba* extract (EGb 761) and its main component ginkgolide A were shown to alleviate paralysis and improve chemotaxis behavior in CL2355 strain. This was attributed not only to their antioxidant properties but also to their ability to directly interact with and inhibit Aβ oligomerization [34]. Similarly, flavones such as scutellarein and baicalein, used in Traditional Chinese Medicine, and some phenolic compounds, like hydroxytyrosol, present in foods typical of the Mediterranean diet (e.g., virgin olive oil), have been demonstrated to induce a notable increase in the chemotaxis index, improving neural function and suggesting a strong potential for clinical applications in Alzheimer’s disease [45]. Moreover, carnosic acid, a polyphenol from rosemary, was found to enhance neurotransmission efficiency and mitochondrial health in *C. elegans*, further confirming the broad neuroprotective effects of polyphenols against Aβ-induced toxicity [50]. These findings collectively emphasize the diverse mechanisms through which polyphenols can mitigate Alzheimer’s pathology, highlighting their therapeutic potential beyond mere antioxidant activity.

The results obtained herein in the *C. elegans* mutants used as AD models revealed that treatment with GSPE significantly increased average and maximum worm lifespan, being able to delay the onset of Aβ-induced paralysis in strains expressing Aβ in muscle (i.e., CL4176 and CL2006), and improving the chemotaxis behavior in worms expressing Aβ in neurons (i.e., CL2355). This suggested that GSPE has the potential to provide in vivo protection against amyloid toxicity in both muscular and neuronal cells, thus offering potential benefits for Alzheimer’s disease. Of particular significance are its effects on neurons, as in AD the small oligomeric forms of Aβ-peptide are regarded as the primary toxic entities affecting these cells [51]. Similar findings on the effect of a polyphenol-rich grape seed extract on slowing the progression of Aβ were made by Wang et al. [9] in a mouse model. Those authors observed a 33% decrease in the total brain Aβ burden in mice fed a diet including the GSP extract compared to those consuming a control diet. Grape seed extracts were also shown to possess Tau and Aβ anti-aggregation properties in mice models of AD [9,52]. In the same way, using synthesized peptides containing the first Proline-rich region of Tau, Gueroux et al. [53] demonstrated that epigallocatechin-3 gallate (EGCG) was able to prevent Tau aggregation by binding to its phosphorylation region and inducing conformational changes in the protein. On the other hand, the oral administration of a grape seed extract to rats was found to increase brain levels of phenolic acids from the bacterial metabolism of flavonoids (e.g., 3-hydroxybenzoic acid and 3-(3′-hydroxyphenyl)propionic acid), which strongly interfered with the association of β-amyloid peptides [21]. Similarly, protocatechuic acid, another bacterial metabolite of flavan-3-ols, was also described to inhibit the aggregation of Aβ and α-synuclein (αS) and destabilize their preformed fibrils in PC12 rat pheochromocytoma cells [54], while catechin and procyanidin A2 were found to inhibit Aβ-induced apoptosis and suppress inflammation in BV-2 cells (an immortalized mouse microglia cell line) through the downregulation of the NF-κB pathway [55].

The increased aggregation and misfolding of proteins, due to the impaired functioning of proteostasis mechanisms, is a key feature of AD and responsible for amyloid-β accumulation. As a part of the proteostasis system, protein degradation mechanisms are triggered when there is a need to reduce the cellular levels of the overall and damaged proteins, which in eukaryotes relies on the proteasome. The impairment of proteasome function results in decreased clearance of target proteins, which has been related to the onset of neurodegenerative diseases [56]. Some polyphenols were also indicated to inhibit the toxic aggregation of amyloidogenic proteins targeting proteostasis [42,43]. Oppositely, in the present study it was observed that despite the mitigation of the toxicity induced by Aβ in the GSPE-treated worms, no enhancement but rather a reduction in the proteasomal activity was produced. This unexpected finding might be explained by the GSPE induction of alternative pathways for the elimination of dysfunctional proteins aside from proteasomal activity. Such pathways may involve protein refolding in the endoplasmic reticulum, mitochondrial unfolding protein response, chaperone activation, or protein degradation through autophagy [57,58]. Thus, exposure to GSPE might be activating endogenous defense mechanisms against oxidative damage, making the worms less reliant on further protein degradation. Our earlier research supports this possibility. It showed that treatment with epicatechin, a major component of the GSPE, reduced oxidative damage in wild-type *C. elegans* under stress. This effect was associated with the overexpression of genes involved in stress resistance, such as *gst-4*, *hsp-16.2*, and *hsp-70* [59].

Autophagy is a crucial mechanism in the development and progression of AD. It is essential for cellular homeostasis and survival, responsible for the degradation and recycling of damaged proteins and organelles, and plays a key role in the cellular response to stress and the prevention of pathological protein accumulation. In AD, dysfunction in autophagy mechanisms is intrinsically linked to the anomalous accumulation of beta-amyloid and Tau protein. It has been shown that the efficiency of autophagy is impaired in the brain of AD patients, in particular due to a lack of lysosomal acidification, which hinders normal autophagic flux and contributes to proteolytic failure. This impaired autophagic function may drive disease progression by allowing the accumulation of toxic protein aggregates and dysfunctional organelles, highlighting the relevance of this process in the pathogenesis and potential treatment of AD [60,61].

To explore the possible influence of autophagy in the observed effects, the impact of GSPE on the expression of several genes involved in this process (*vha-5*, *cpr-5*, *epg-8*, *imp-2*, and *rab-7*) was studied.

*vha-5* encodes a vacuolar (H+)-ATPase involved in maintaining lysosomal acidity and ensuring efficient degradation processes [62]. Similarly, *cpr-5* is the ortholog of human cathepsin B (CatB), a lysosomal cysteine endopeptidase that facilitates cellular proteolysis [63]. *epg-8* is associated with the phosphoinositide 3-kinase (PI3K) complex, and studies have shown that reducing autophagosome levels via the PI3K complex can delay Aβ-induced paralysis and mitigate Aβ-related toxicity in the CL4176 strain, providing neuroprotective effects [64]. The *imp-2* and *rab-7* genes are called autophagosome–lysosome fusion genes because of the important role they play at this junction [64,65]. Successful fusion requires that autophagosomes encounter late lysosomes or endosomes. The effector proteins of the RAB proteins include motor protein-binding adaptors that are able to direct the transport of these autophagosomes along the cytoskeleton [65].

As described in Figure 6, only the *imp-2* gene had a significantly increased expression in the worms treated with GSPE compared to the controls, suggesting an enhanced efficiency of the autophagosome–lysosome fusion. IMP-2 affects the stability or translation of mRNAs encoding key components of the autophagic machinery, such as ATG proteins, LC3, or lysosomal enzymes, thus playing an indirect but critical role in the modulation of autophagy. For example, it could stabilize mRNAs encoding proteins essential for autophagosome initiation and expansion, or even influence the cell’s response to stress signals that activate the autophagic pathway [66,67]. Thus, the observed increase in *imp-2* expression could improve the efficiency of the autophagy process and, therefore, result in more effective removal of damaged cellular components in the GSPE-treated animals, helping protect against the toxicity induced by the Aβ peptide accumulation.

Other observations have, however, been made in different studies where the effects of distinct flavan-3-ols were explored. In a previous study, we observed that epicatechin increased the expression of *cpr-5* but decreased that of *epg-8* in CL4176 worms [41]. This suggested increased proteolysis but decreased autophagosome accumulation. Other authors have reported that EGCG could induce the lysosomal degradation of cellular components, which may be related to autophagy [68]. Devika et al. [69] noted that EGCG also stabilizes lysosomal enzymes, likely supporting degradative processes within the autolysosome. Recently, EGCG was shown to be able to directly target intracellular Aβ aggregates and promote their lysosomal degradation [70]. All in all, it seems evident that different flavan-3-ols may show distinct mechanisms of action and differentially influence the expression of genes involved in the autophagy/lysosomal system. More studies are needed to clarify the specific mechanisms through which polyphenols, either as individual compounds or in mixtures, act at the molecular level, as well as to improve the knowledge about their bioavailability, in which the intestinal microbiota can play a key role. This understanding is essential for their potential use in the prevention of neurodegenerative disorders or other chronic diseases.

## 5. Conclusions

This study demonstrates that the studied grape seed polyphenol extract induced significant neuroprotective properties in *Caenorhabditis elegans* models, alleviating amyloid-β peptide-induced toxicity. The studies carried out demonstrated that the grape seed polyphenol extract effectively reduced amyloid-β peptide accumulation in strain CL4176, a *C. elegans* model of Alzheimer’s disease, resulting in a remarkable decrease in the rate of paralysis. Furthermore, the grape seed polyphenol extract significantly increased the longevity of the CL2006 strain, which expresses human Aβ₁₋₄₂ in muscle cells, and also improved the sensory response of the CL2355 strain, which expresses Aβ in neurons. These observations suggest that grape seed polyphenols could protect against amyloid-β-induced neuronal deficits, thereby improving neuronal function and survival. A reduction in proteasomal activity was also observed in the treated worms, suggesting that fewer misfolded proteins required degradation in the presence of grape seed polyphenol extract, which implies that it may help promote correct protein folding or more efficient elimination of misfolded proteins before they accumulate toxically. Additionally, changes in the expression of the autophagy- and proteostasis-related gene *imp-2* support the notion that grape seed polyphenol extract exerts protective effects by modulating the cellular machinery for protein handling and oxidative stress. On the whole, these observations suggest that grape seed polyphenols could interfere with amyloid-β aggregation and/or promote its degradation, possibly by influencing the cellular processes involved in protein management and stress response, among other mechanisms. All in all, these promising findings support the hypothesis that polyphenols, such as those present in grape seeds, could provide effective protection to prevent or slow the progression of neurodegenerative diseases. Further studies are, however, required to fully elucidate the molecular mechanisms underlying these neuroprotective effects. Future research should also explore the bioavailability and efficacy of these compounds in more complex in vivo models.

## Figures and Tables

**Table 1:** Oligonucleotide primers used in RT-qPCR studies.

Gene	Forward	Reverse
vha-5	CTTCATGGAAACGCGACTGT	CGGTAACGAACACCATGTGC
*cpr-5*	CTCCGACGCTATTCCAGACC	GCGTAGGCGGTAGATCCAAA
*epg-8*	GCGGTAAACGCTACACAAAGA	CCATCCGCTGAGATTCCTGG
*imp-2*	CGCTGCTTTTGAAGCCTTGT	AGTGTCCGCTGTTGAGGATG
*rab-7*	TTCCTCACACGCGACGTAAA	CGGCTCCACGATAAAAAGCG
*act-1*	CCAGGAATTGCTGATCGTATG	GGAGAGGGAAGCGAGGATAG

**Table 2:** Mass spectral data and tentative identification of the peaks in Figure 1.

Peak	Pseudomolecular Ion [M-H]^−^ (*m*/*z*)	MS^2^ Fragment Ions (*m*/*z*)	Tentative Identification
1	577	425, 407	Procyanidin dimer B3
2	577	425, 407	Procyanidin dimer B1
3	865	577, 425, 407	B-type procyanidin trimer
4	577	425, 407	Procyanidin dimer B4
5	577	425, 407	Procyanidin dimer B2
6	289	-	Epicatechin
7	729	577, 289	Galloyled procyanidin dimer
8	577	425, 407	Procyanidin dimer B5
9	729	577, 289	Galloyled procyanidin dimer
10	729	577, 289	Galloyled procyanidin dimer
11	729	577, 289	Galloyled procyanidin dimer

**Table 3:** Quantification of flavan-3-ols in the grape seed polyphenol extract.

Compound	Concentration * (mg/g)
Epicatechin	14.80 ± 0.23
B-type procyanidin dimers	69.65 ± 0.35
Galloyled procyanidin dimers	40.53 ± 0.31
B-type procyanidin trimer	37.85 ± 0.28
Total flavan-3-ols	162.83

* expressed as epicatechin equivalents.

**Table 4:** Lifespan of *C. elegans* CL2006 cultivated at 20 °C with or without GSPE (100 µg/mL) added to the culture medium.

Assay	Mean (Days) ^1^	*p* vs. Control (Log Rank)	Maximum 10% (Days) ^2^	*p* vs. Control (ANOVA)
Control	11.99 ± 0.44		19.10 ± 0.79	
GSPE (100 µg/mL)	13.69 ± 0.42	0.009	20.70 ± 1.34	0.009

^1^ data are presented as mean ± standard deviation from three independent experiments (n = 100 worms per experiment). ^2^ maximum lifespan was calculated as the average lifespan of the top 10% longest-living individuals in each group. Statistical analysis was performed using the log-rank test for mean lifespan and one-way ANOVA for maximum lifespan, with significance set at *p* < 0.05 for both analyses.

## Data Availability

The original contributions presented in the study are included in the article, further inquiries can be directed to the corresponding author.
